# Supplementary material for: Posttranslational Modification of Human Cytochrome CYP4F11 by 4-Hydroxynonenal Impairs ω-Hydroxylation in Malaria Pigment Hemozoin-Fed Monocytes: The Role in Malaria Immunosuppression
Source: Int J Mol Sci. 2023 Jun 16;24(12):10232. doi: 10.3390/ijms241210232 (PMC10299294; doi:10.3390/ijms241210232)
Supplement: Supplementary file 1 [file ijms-24-10232-s001.zip › ijms-2422999-supplementary.pdf]

<u>Supplemental Data</u> for Skorokhod et al. "Posttranslational modification of human cytochrome CYP4F11 by 4-hydroxynonenal impairs ω-hydroxylation in malaria pigment hemozoin-fed monocytes: the role in malaria immunosuppression"

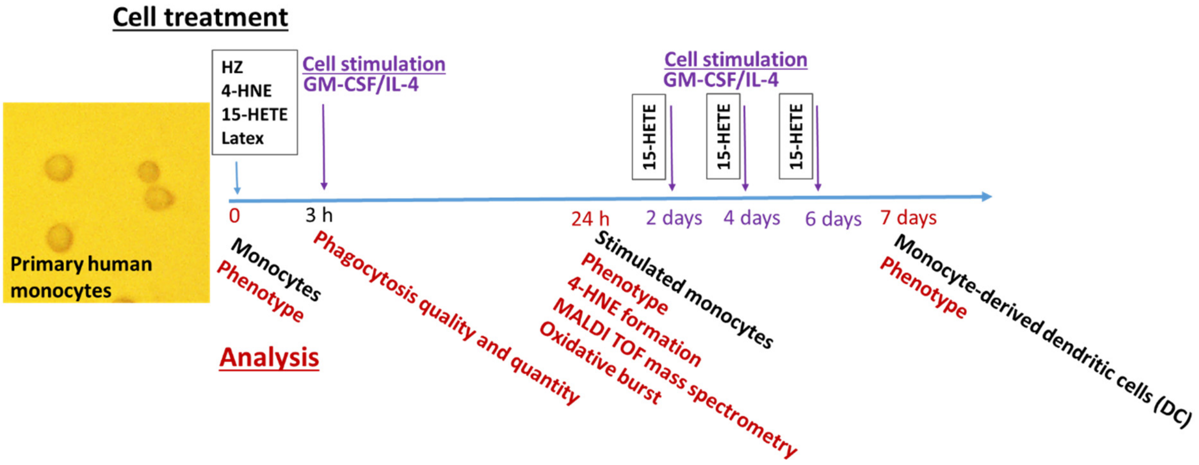

Supplemental Figure S1. Scheme of cellular study experimental design. Adherent human monocytes were supplemented with hemozoin (HZ), 4-hydoxynonenal (4-HNE), 15-hydroxyeicosatetraenoic acid (15-HETE) or neutral latex beads (Latex). The phagocytosis was completed in 3 h, when non-phagocytosed material was washed out and the incubation medium was changed with supplementation of 80 ng/ml granulocyte macrophage colony-stimulating factor (GM-CSF) and 40 ng/ml interleukine-4 (IL-4). Performed analysis are indicated at respective points below the timeline. The applied mass spectrometry technique was MALDI-TOF (matrix-assisted laser desorption/ionization and time of flight detection).

Monocyte plating procedure: Two millilitres of monocytes, suspended at 10 mln cells per millilitre of RPMI 1640 cell culture medium supplemented with L-glutamine, were plated in each 35 mm diameter culture dish. After a 30 min incubation at 37°C, dishes were washed three times with RPMI 1640 to remove non-adherent cells. Two millilitres of macrophage serum free medium (MSFM, Gibco, ThermoFisher) was added to each dish.

HZ phagocytosis assessment: the phagocytosis was assessed in one of the replicate dishes with HZ-fed cells and compared to controls in bright field microscopy at 3h and 24h (Leica DR IRB fluorescence microscope equipped with a Leica DFC 420C camera, a 63× oil planar apochromatic objective with 1.32 numerical aperture, version 3.3.1 of the Leica DFC image software (Leica Microsystems, Wetzlar, Germany)).

Cell phenotyping: monocyte and dendritic cell phenotype was characterised in terms of CD1a, CD14, CD40, CD54, CD64, CD83, MHC class II (all antibodies were from BD Biosciences) antigen expression by flow cytometry (FACS; FACSCalibur cytofluorograph and CellQuest software, BD Biosciences).

4-HNE conjugate assessment: monocytes were washed in PBS, supplemented with bovine serum albumin at 2% w/v (PBS-A), and incubated with saturating concentrations of anti-4-HNE monoclonal mouse primary antibody (1:50 dilution) at room temperature for 1 h. After three washes in PBS-A, bound antibodies were revealed by FITC-conjugated anti-mouse IgG secondary antibody (1:300 dilution) by FACS.

Monocytes were differentiated to DC during 7 days by supplementation with 80 ng/ml GM-CSF and 40 ng/ml IL-4 every 48 h during medium change, as shown in the diagram. Performed analysis are indicated at respective points below the timeline (non-linear).

BLAST alignment (https://blast.ncbi.nlm.nih.gov/Blast.cgi), query **human CYP4F11, Sbjct mouse CYP1A2**

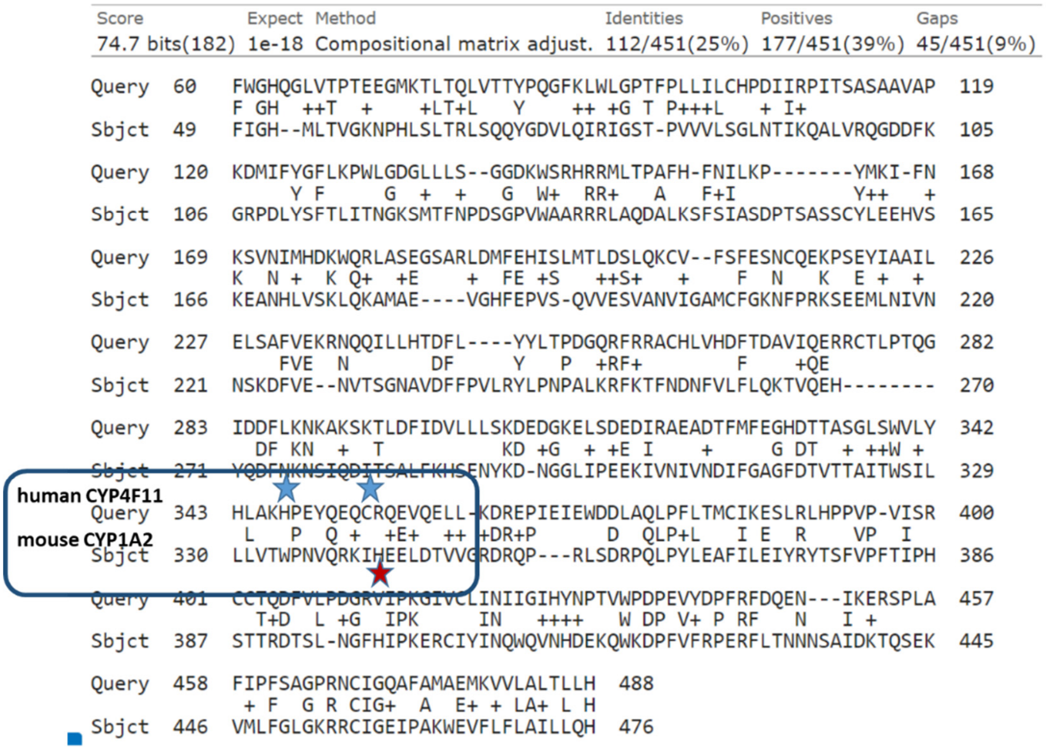

Supplemental Figure S2. BLAST alignment of human CYP4F11 and mouse CYP1A2 cytochromes. Blue asterisks are 4-HNE modified residues histidine H347 and cysteine C354 from human CYP4F11 found in this study, red asterisk is mouse CYP1A2 histidine H342 in mouse CYP1A2 described before [Golizeh et al. Identification of 4-hydroxynonenal protein targets in rat, mouse and human liver microsomes by two-dimensional liquid chromatography/tandem mass spectrometry. *Rapid Commun Mass Spectrom.* 2016;30(13):1488-94].
